# Epidemiology of violence and suicide risk in Senegal: A nationwide survey in 2023

**DOI:** 10.1371/journal.pgph.0005782

**Published:** 2026-01-02

**Authors:** Jean Augustin Diégane Tine, Véronique Petit, Mbayang Ndiaye, Hélène Langet

**Affiliations:** 1 Mental Health Division, Ministry of Health and Social Action, Dakar, Senegal; 2 Institute of Health and Development (ISED), Cheikh Anta Diop University (UCAD), Dakar, Senegal; 3 UMR 196, Center for Population and Development (CEPED), Institute for Research on Development (IRD), University of Paris Cité, Paris, France; 4 Swiss Tropical and Public Health Institute, Allschwil, Switzerland; 5 University of Basel, Basel, Switzerland; McGill University, CANADA

## Abstract

Assessing violence and suicide risk at the population level is essential to inform public health policies and guide prevention efforts for mental health. In Senegal, as in much of sub-Saharan Africa, such data remain scarce. This study aims to fill that gap by estimating the prevalence of violence and suicide risk, and identifying associated factors in the Senegalese population. This nationwide, observational, cross-sectional, population-based analytical survey received ethical approval from Senegal’s National Ethics Committee for Health Research. Conducted between July and August 2023, it covered 496 randomly selected households proportionally distributed by demographic zone. Participants were surveyed on their exposure to different forms of violence, help-seeking behaviours, suicidal ideation and behaviours, and psychiatric history. Data were collected via ODK and analysed in R. Out of 2174 respondents (33.58% youth, 49.95% adults, 16.47% elderly), 52.76% reported exposure to violence, predominantly psychological (47.38%), verbal (43.47%), and physical (32.84%). Co-occurrence was frequent: 37.14% reported combined psychological and physical violence. Age and marital status were strongly linked to violence exposure, with women being more exposed to sexual violence (OR = 1.75 [1.12–2.80]). Dakar was identified as the main violence hotspot. The overall suicide risk was 8.40%, with 1.66% at high risk. Exposure to any form of violence significantly increased suicide risk. Individuals diagnosed with a mental illness were at higher risk (OR = 4.76 [3.13–7.14]). Despite high violence prevalence, support remains rare: only 6.2% received psychological help, 10.4% police assistance. Findings reveal that violence and suicide risk are widespread but insufficiently addressed. The results call for urgent development of a national mental health policy centred on prevention and support for vulnerable groups, especially in the current socio-economic and environmental crisis context.

## Introduction

Violence is a recurring theme in the media coverage, with rape, verbal violence, genital mutilation and domestic violence being frequently reported. However, beyond the stories and images that are disseminated *ad nauseam* and often without adequate ethical framing, the epidemiological, socioeconomic and anthropological aspects of the various forms of violence as well as their morbid, social and legal consequences need to be better documented, whether they occur within families, communities or disproportionately affect specific population groups [1]. Suicide, a sub-category of violence, claimed approximately 703,000 lives worldwide in 2019, according to the World Health Organization (WHO). This accounts for 1.3% of all deaths, 77% of which occurred in low- and middle-income countries [2]. Although suicide can occur at any stage of life, it is the fourth leading cause of death among young adults aged 15–29. The most common methods used are hanging, self-ingestion of pesticides and firearms [3,4]. In addition to its health and mortality consequences, suicide carries considerable and lasting psychosocial and economic costs for individuals, families and communities [5]. Although suicidal thoughts and behaviours are recognized as significant public health concerns, progress in reducing suicide rates has remained limited over the past decades [6,7]. As part of the global effort to address this issue, the suicide mortality rate serves as a key indicator for monitoring progress toward Target 3.4 of the Sustainable Development Goals (SDGs), which aims to reduce premature mortality from non-communicable diseases (NCDs) by one-third by 2030 through prevention and treatment [8]. Few countries however have high-quality data on suicides and suicide attempts, and even fewer have defined a national suicide prevention strategy [9]. According to WHO, “*suicide rates tend to be underestimated because of inadequate surveillance systems, or because suicides are attributed to accidental deaths, or because they are criminalized in some countries*” [10]. One critical step toward effective prevention and treatment of suicidal thoughts and behaviours is to identify risk factors, e.g., longitudinal predictors [9]. A history of trauma or exposure to violence is among the factors contributing to an increased risk of suicide and suicidal ideation [7,11].

In the WHO Africa region, the suicide rate is higher than the global average (11.2 versus 9.00 per 100,000 people in 2019), and six African countries are among the ten countries with the highest suicide rates worldwide [2]. In many traditional African cultures, the fear of death by suicide is very present, constituting one of the strongest taboos. In East Africa, for instance, suicide is considered a terrible event for family and close friends [12]. Nonetheless, some types of violence, particularly those based on gender (domestic and sexual violence, widowhood rites, levirate, early and forced marriages, female genital mutilation, rape during conflict) have acquired greater visibility in the public arena due to democratization, feminist struggles and the appropriation of the culture of law [13,14]. Even traditionally taboo subjects such as suicide are increasingly publicly discussed [15]. Despite this growing public awareness, violence has not yet been fully recognized as a public health priority in sub-Saharan Africa. One major barrier is the general scarcity of population-level data, which limits the ability to investigate the risk factors associated with violence and suicide in the region – with the exception of South Africa, where comparatively more data are available, though substantial gaps remain [16,17].

Senegal, a country in the westernmost part of Africa, bordered by Mauritania, Mali, Guinea and Guinea-Bissau, is no exception, and is characterized by insufficient statistical data on violence and mental health. Various reports point to the inadequacy of resources and public policies devoted to combating NCDs and improving the mental health of the population. Available studies focus on drawing up an epidemiological, clinical or judicial assessment of physical violence against women at a regional level, such as in Kolda [18], Tambacounda [19] or in several regions [20]. The authors emphasize the intensity of the violence suffered, even if the question of its measurement based on declarative data remains open: “*Violence against women and girls remains significant in Senegalese society. However, the main problem lies in the lack of reporting of the harm suffered by these victims. The atrocity of the violence, with negative repercussions on the physical, mental and social levels of the victims, must encourage the authorities to include actions to combat violence against women and girls in public health policy strategies*” [20]. Meanwhile, the 2023 field visit report in Senegal by UNICEF and its partners highlights “*the multiplication of non-communicable diseases*” and the plurality of violence: “*around 10.4% of women aged 15 to 49 have experienced physical violence and 3.4% sexual violence. One in three women aged between 20 and 49 (32%) is married before the age of 18. A very large number of children are exploited, boys through forced begging and girls through domestic work and sexual exploitation. Between 2017 and 2019, the proportion of girls aged 10 to 14 who have undergone FGM rose from 18% to 20%*.” [21].

This research aims to gain a better understanding of the epidemiological realities associated with violence and suicide risk in Senegal. More specifically, it seeks to (i) assess the prevalence of different forms of violence, (ii) assess the prevalence of suicide risk, (iii) describe the socio-demographic and clinical characteristics associated with suicide risk, and (iv) analyse the relationships between forms of violence and suicide risk in the Senegalese population.

## Methods

This study is the result of a national survey on the mental health of the Senegalese population, which took place from July 24 to August 6, 2023. The survey was funded by UNICEF and conducted in partnership with the Mental Health Division of the Ministry of Health and Social Action (MSAS) and the National Agency for Statistics and Demography (ANSD) of Senegal.

### 1.1. Settings

According to the 2023 national census, Senegal has a population of 18,032,473, predominantly Muslim, with a multi-ethnic mix of Wolof, Serer, Pulaar and Diola [22]. The country is characterized by its youthfulness, with half of the population under 19 and 39.2% under 15. The sex ratio is 102.6 men for every 100 women overall, with notable variations across age groups: 110.8 among those under 15, dropping to 98.1 among those aged 15–60, and to 93.5 among those aged 60 and above. The population is unevenly distributed across the country, with the highest concentrations in the west, centre and northwest, along the Dakar-Thiès-Diourbel axis. Between 2013 and 2023, population density rose from 65 to 92 inhabitants/km^2^, with the capital Dakar standing out with a density of 7277 inhabitants/km^2^. The average household size is nine people, ranging from six in Dakar to twelve in the regions of Tambacounda, Sédhiou, Matam, Kaolack and Kaffrine. Among households, 45.6% are engaged in farming.

### 1.2. Design and sampling

This study is a cross-sectional, observational survey conducted at the household-level, using a three-stage proportional sampling design: (i) a stratified draw of communes, (ii) a random selection of census districts based on the ANSD national directory, and (iii) the systematic drawing of households to be surveyed in the census district. In each census district, six households were selected using the itinerary method, with the sampling step calculated as the number of concessions in the census district divided by six. Up to six individuals were selected from each selected household based on their inclusion in one of six pre-defined age groups: 5–14, 15–18, 19–23, 24–39, 40–59, and 60 years and older. Once inside the household, the interviewer enumerated all household members and then randomly drew lots according to age. The sample size was determined using the STEPS survey methodology and associated parameters [23], resulting in a minimum sample size of 1,843 individuals. Details of the calculation are provided in the supporting information (S1 Text).

### 1.3. Measures: definitions and operationalization

The survey questionnaire was designed by a multidisciplinary team of researchers specializing in mental health and demography in Senegal, drawn from the project’s supporting institutions and their scientific partners. It covered a range of topics, including experience of various forms of violence, suicide risk, neuropsychiatric disorders, and assistance-seeking behaviour.

#### Forms of violence.

**Fig 1:**
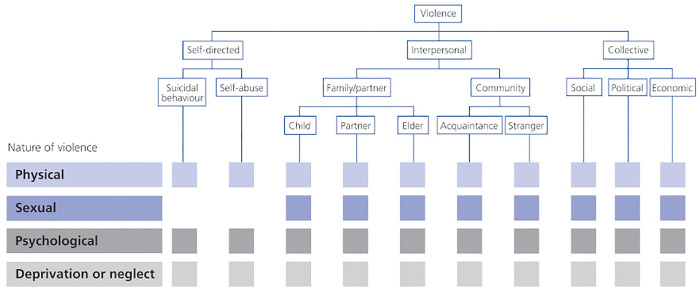
WHO typology of violence. Reproduced from the World Report on Violence, 2002 [25].

The inherent complexity of the phenomenon of violence makes it difficult to establish a unanimous definition. As a result, violence is frequently interpreted in different ways by different people and in different contexts. Irrespective of the scale of countries, cultures or belief systems, violence remains a social fact that can take on contrasting textures ranging from the physical to the symbolic [24]. The study adopted WHO’s definition from the 2002 World Report on Violence and Health: “*the intentional use of physical force or power, threatened or actual, against oneself, another person, a group or a community, that either results in or has a high probability of resulting in injury, death, psychological harm, maldevelopment or deprivation*” [1,25]. This definition associates intentionality with the act of violence itself, regardless of its outcome. It therefore excludes unintentional incidents, such as most road accidents and burns. Violence is classified into three broad categories according to the characteristics of the perpetrators: self-directed violence, of which suicide is a sub-category, interpersonal violence, and collective violence. On the one hand, this definition makes it possible to include all types and forms of violence, irrespective of the background or stage of life of the people concerned. On the other hand, it makes explicit the consequences of violence on the physical and mental health of the person subjected to it. Each type of violence can take several forms. The forms of violence refer to the nature of the acts. The most commonly considered forms are physical violence, sexual violence, psychological and verbal violence, and deprivation or neglect. Other forms of violence are specific to particular issues, such as economic violence in a marital context, or elder violence (Fig 1).

Participants were asked whether they had ever experienced acts of violence that had affected them significantly. For those responding positively, additional information was collected on the nature of the violence and the identity of the perpetrator(s). Responses on the nature of violence were categorized into predefined types (e.g., exclusion, discrimination, stigmatization, sexual violence). Additional follow-up items were included to capture specific experiences (e.g., “*Has someone ever attacked, assaulted, or hit you at home or in your neighborhood?*”; “*Has someone ever insulted, intimidated, harassed, or threatened you at home or in your neighborhood?*”). Survey items were subsequently grouped according to the conceptual definitions outlined in Table 1.

**Table 1:** Conceptual definitions of forms of violence used in the survey.

Form of violence	Definitions
Psychological violence	Psychological violence is malicious and harmful behaviour that aims to control, intimidate or manipulate a person without resorting to physical acts. Psychological violence can include humiliation, intimidation, verbal threats, harassment, denigration, emotional manipulation, excessive control, social isolation and other tactics designed to exert psychological control over the victim. Among specific forms of psychological violence, stigmatization, involves labelling, discrediting, or marginalizing a person or group based on an attribute perceived as deviant, while discrimination refers to unequal or prejudicial treatment directed at individuals due to their real or perceived membership in a particular social group.
Verbal violence	Verbal violence refers to a range of verbal acts including abusive, humiliating, threatening or degrading language that causes harm and emotional suffering.
Domestic violence	Domestic violence refers to a series of abusive and violent behaviours that occur within an intimate family relationship or with a loved one. It takes different forms: physical violence, psychological violence, sexual violence, economic violence, coercion. Domestic violence occurs between spouses or intimate partners, family members, cohabitants, ex-spouses, parents and children. Domestic violence can also include domestic harassment, domestic assault, and beatings.
Sexual violence	Sexual violence is any non-consensual, abusive act or behaviour of a sexual nature perpetrated against a person. These acts include a variety of behaviours ranging from sexual assault to rape and other forms of sexual violence.
Physical violence	Use of physical force against another person or group of people, resulting in physical, sexual or psychological harm.
Neglect	Neglect is when the person responsible for the child (parents, grandparents, etc.) deprives him or her of the elements essential to proper development and well-being (deprivation of food, sleep, care and attention). Neglect is thus a form of violence by omission, i.e., the failure to mobilize the adult on whom the child’s present and future depend. It is characterized by its chronic or repeated nature. Invisible and often overlooked, neglect nevertheless affects the child’s survival, security, awareness, self-esteem and education.
Economic violence	Economic violence refers to all abusive, coercive or negligent behaviour aimed at depriving a person of their financial resources, economic autonomy or basic subsistence rights. Such abuse can be direct (food deprivation, denial of access to money, total control of income) or indirect (financial blackmail, forced indebtedness, exploitation of dependency).

#### 1.3.1. Assistance-seeking behaviour.

Assistance-seeking behaviour following episodes of violence can be defined as any action taken voluntarily by an individual victim of violence to access (i) medical assistance, which corresponds to any consultation, care or hospitalization, (ii) psychological assistance, which includes therapeutic support received from psychologists, social workers, or other mental health professionals, and (iii) police alert, which includes contacting law enforcement, filing a complaint, or seeking a legal protection. In the survey, assistance-seeking behaviour was assessed using a five-point Likert-scale with questions measuring how frequently individuals sought each type of assistance following episodes of violence.

#### Suicide risk assessment.

The survey also examined suicide risk, recognizing that individuals who are victims of violence – whether physical, sexual, psychological or negligent – are more likely to present an increased risk of suicidal thoughts and behaviour, even if powerful protective factors exist such as social support, a community mental health system, etc. Suicide is defined as the act of deliberately taking one’s own life. It includes suicidal ideation, planning and attempts. Suicide can be analysed as “*the result of an accumulation of interacting individual, relational and community risk and protective factors*” [26]. Traumatic experiences linked to the nature of the type of violence can have a profound impact on the mental health of individuals, contributing to an increase in suicide risk, which corresponds to the probability that a person will attempt suicide or cause serious injury to themselves. Suicide risk is an important indicator in the prevention of suicide attempts. Suicide risk was assessed using the suicidality module of the Mini International Neuropsychiatric Interview (MINI) Version 5, which includes six items covering suicide ideation, planning, and attempts, and stratifies respondents in four risk categories: no risk, low risk, moderate risk and high risk [27].

#### Neuropsychiatric disorders.

Neuropsychiatric disorders were operationally defined as all mental or neurological disorders affecting an individual’s behaviour, cognition, emotions or functional abilities, diagnosed or suspected based on observation. Participants were first asked: *“Do you have a history or current condition of psychiatric or neurological disorders?”* If the response was affirmative, additional information was collected on diagnosis, onset, treatment, and care providers. Where possible, medical documentation (e.g., psychiatric prescriptions, medical certificates) was requested. For respondents unable to answer due to age, disability, or cognitive impairment, proxy responses were obtained from legal guardians, household heads or care attendants. The presence of symptoms compatible with a neuropsychiatric disorder (e.g., behavioural disorder, disorientation, hallucinations, emotional instability, mutism) observed by the interviewer was also considered an indicator of suspected disorder in the absence of documentation. This approach made it possible to reconcile the epidemiological imperatives of data validity with field realities (absence of medical records, low mental health literacy), while ensuring respect for the dignity of interviewees.

### 1.4. Data collection and analyses

Data collection was carried out at designated survey sites by a team of twenty interviewers, including eight women. Prior to fieldwork, all interviewers completed a five-day training on the administration of the questionnaires and were sensitized to mental health issues by experts from the technical committee overseeing this research. All interviewers were trained to ensure that no moral, religious or social judgments were made about certain forms of violence that may be taboo/sensitive, such as attempted suicide or domestic and sexual violence. A pre-test of the questionnaire was carried out in the field to refine the tools and expose interviewers in real data collection scenarios. Interviews were conducted in French or in local languages (Wolof, Serer, Halpulaar, or Diola) according to the language skills of the interviewees. Data collection took place in July and August 2023 using the ODK Collect application.

Analyses were carried out using the R software, within the Rstudio/Quarto interfaces, and the following R packages: dplyr, gtsummary, ggplot2, rnaturalearth, and ComplexUpset [28–32]. Results were summarised using descriptive statistics. For analytical purposes, the original six age groups from the survey were consolidated into three categories: youths aged 5–18 years, adults aged 19–59 years, and elderly individuals aged 60 years and older. Categorical variables were presented as counts and percentages (n, %), with 95% confidence intervals (CIs) provided where relevant. Associations between socio-demographic factors and violence were assessed using Pearson’s Chi-squared test, with statistical significance determined by two-sided p-values. For associations reaching statistical significance (p < 0.05), odds ratios (ORs) with 95% CIs were estimated using logistic regression models. A multivariable logistic regression model was fitted to examine the association between suicide risk and neuropsychiatric disorders, with results reported as ORs with 95% CIs.

### 1.5. Ethical considerations

The study was conducted in full compliance with the ethical principles outlined in Senegal’s code of ethics. Ethical approval was granted by Senegal’s National Ethics Committee for Health Research (n°SEN23/31/CNERS-19/06/2023) [33]. Authorization from the head of each household was requested before enumerating its members and before approaching any individual selected to participate in the survey, to ensure trust and cooperation. Written informed consent was obtained and documented from all survey participants or their legal guardians. To guarantee privacy and allow participants to express themselves as freely and confidently as possible, interviews were conducted in strict confidence, at a location of their choosing within the household. Participants were informed that they had the right to withdraw from the interview at any time without justification if they no longer wished to participate. If specific medical needs were identified, interviewers were instructed to inform the individual and/or their relatives about available healthcare options in the area and, where appropriate, to offer a referral to the nearest health facility.

### 1.6. Inclusivity in global research

Additional information regarding the ethical, cultural, and scientific considerations specific to inclusivity in global research is included in the supporting information (S1 Checklist).

## Results

### 1.7. Characteristics of the population

A total of 361 households, comprising 2,174 individuals, were surveyed across Senegal. The sample was nearly gender-balanced, with slightly more women (50.69%) than men (49.31%). The average age was 41.4 years, with a standard deviation of 12.2 years. The age distribution showed a predominance of young people and adults: 33.58% of respondents were aged 5–18 years, 49.95% were aged 19–59 years, and 16.47% were aged 60 years and older. In terms of marital status, 44.25% of respondents were married, 39.01% were single, and 16.74% fell into other categories (e.g., divorced or widowed). The education rate was relatively high, with 73.64% of respondents having received formal primary-level instruction (95% CI: 71.73%-75.47%). Among the respondents who were professionally active, the majority were employed in the formal private sector (75.81%), followed by 14.26% in the public sector and 9.92% in the informal private sector. Most participants reported modest incomes: 61.71% earned less than 100,000 XOF per month, 28.53% earned between 100,000 and 200,000 XOF, and only 9.77% reported earning 200,000 XOF or more (Table 2).

**Table 2:** Socio-demographic characteristics of the survey population.

Individual socio-demographic characteristics	N = 2,174^1^	95% CI
Gender		
Men	1,072/ 2,174 (49.31%)	[47.19% - 51.43%]
Women	1,102/ 2,174 (50.69%)	[48.57% - 52.81%]
Age		
5-18 years old	730/ 2,174 (33.58%)	[31.60% - 35.61%]
19-59 years old	1,086/ 2,174 (49.95%)	[47.83% - 52.08%]
60 + years old	358/ 2,174 (16.47%)	[14.95% - 18.11%]
Marital status		
Married	962/ 2,174 (44.25%)	[42.15% - 46.37%]
Single	848/ 2,174 (39.01%)	[36.95% - 41.10%]
Other	364/ 2,174 (16.74%)	[15.21% - 18.39%]
Instruction		
Yes	1,601/ 2,174 (73.64%)	[71.73% - 75.47%]
No	573/ 2,174 (26.36%)	[24.53% - 28.27%]
Professional sector		
Public	92/ 645 (14.26%)	[11.71% - 17.26%]
Formal private	489/ 645 (75.81%)	[72.28% - 79.03%]
Informal private	64/ 645 (9.92%)	[7.78% - 12.56%]
Monthly income (in XOF)		
<100k	398/ 645 (61.71%)	[57.82% - 65.45%]
100k-200k	184/ 645 (28.53%)	[25.10% - 32.21%]
≥200k	63/ 645 (9.77%)	[7.64% - 12.39%]

^1^n/ N (%). Abbreviation: CI = Confidence Interval. k = thousands (100k = 100,000) 1XOF = 0.0018USD

### 1.8. Violence

#### Overall prevalence of violence.

**Fig 2:**
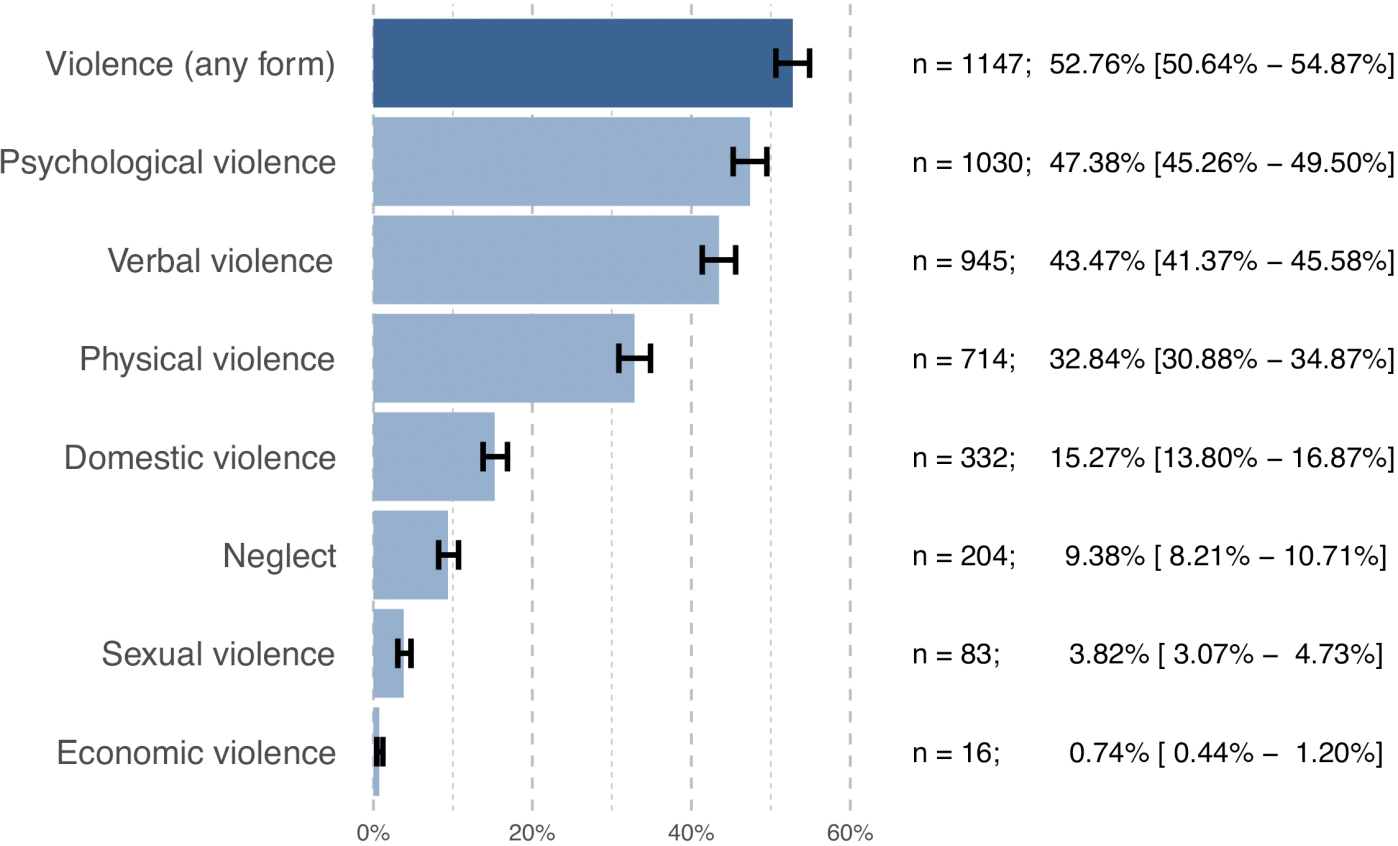
Prevalence (n, %, 95% CI) of the different forms of violence, presented in descending order. The prevalence of any form of violence, i.e., respondents who experienced at least one type of violence, is displayed in a darker blue for visual distinction.

The analysis revealed that more than half of the survey participants (52.76%) had experienced at least one form of violence. Psychological violence emerged as the most common form, affecting 47.38% of respondents, closely followed by verbal violence (43.47%). Physical violence was experienced by nearly one-third of respondents (32.84%), while domestic violence affected 15.27% of the respondents and neglect 9.38%. Although sexual violence was less widespread (3.82%), it remains a concern given its implications. Economic violence was the least frequently reported form of violence, only affecting 0.74% of respondents (Fig 2). Stigma and discrimination, which are specific forms of psychological violence, were reported by 4.83% and 6.26% of participants, respectively. Domestic harassment and domestic assault, which are specific forms of domestic violence, were reported by 12.56% and 8.46% of participants, respectively.

**Fig 3:**
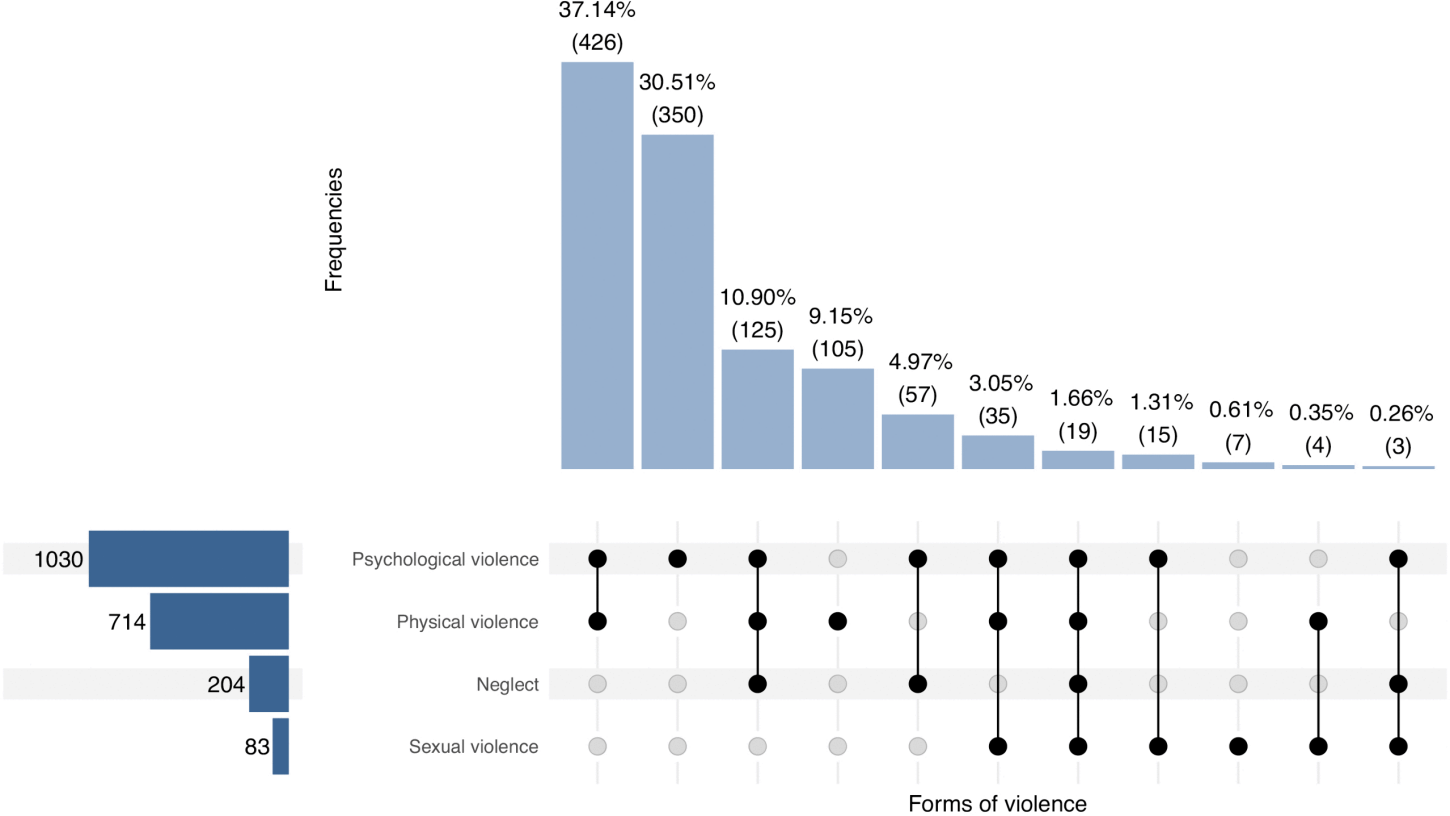
Prevalence of violence in isolated and co-occurring forms, limited to the four main categories defined by the WHO violence typology.

Patterns of polyvictimization – i.e., the experience of multiple, overlapping forms of violence, whether or not occurring simultaneously –, were observed in more than half of survey participants exposed to violence. The most commonly reported exposure to violence was a combination of psychological and physical violence, experienced by 426 individuals (37.14%). A triad of psychological, physical, and neglect-related violence was reported by 125 individuals (10.90%) while psychological, physical, and sexual violence was reported by 35 individuals (3.05%). Notably 19 individuals (1.66%) had reported experience of all four forms of violence: psychological, physical, neglect-related, and sexual (Fig 3).

#### Spatial distribution of violence.

**Fig 4:**
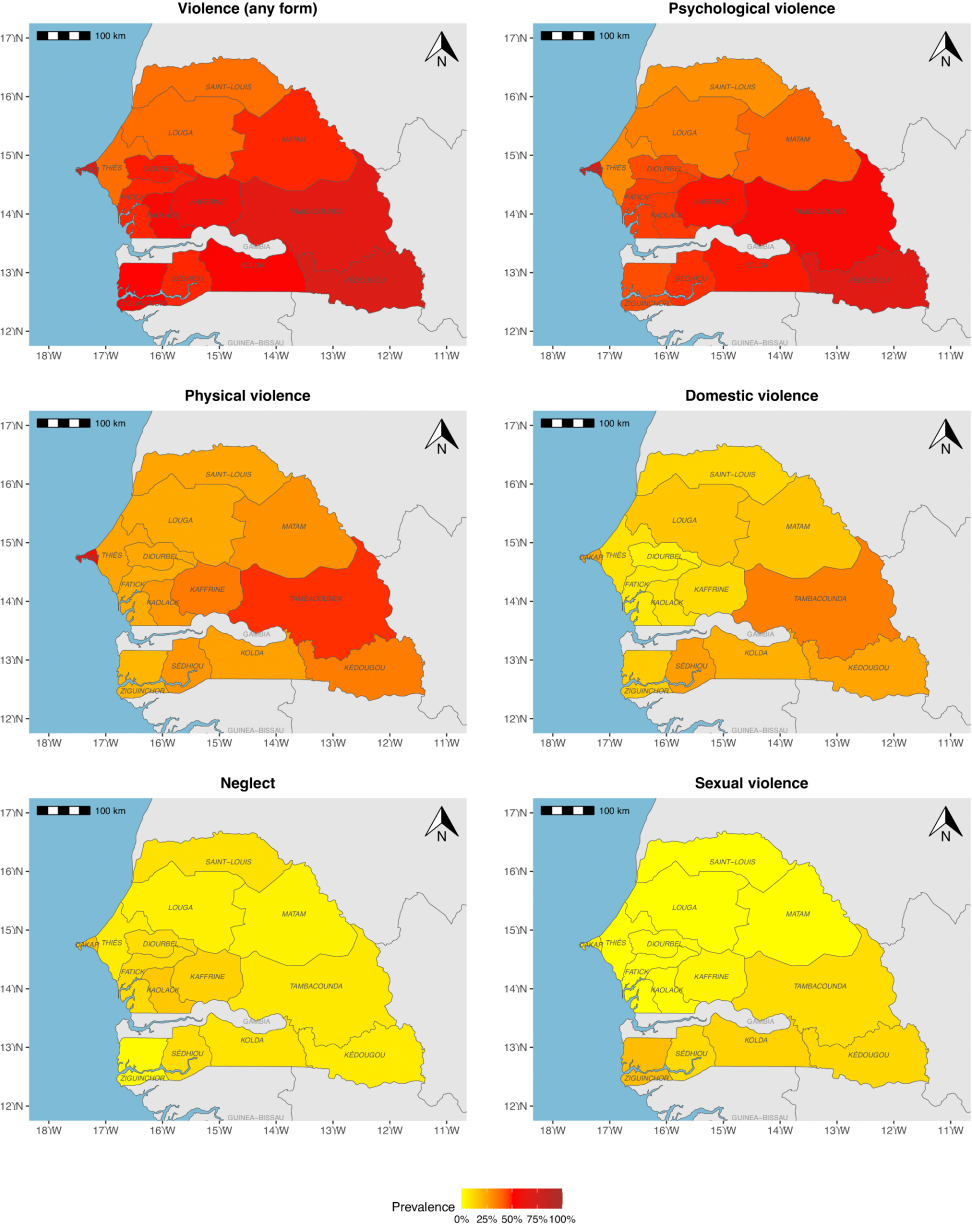
Spatial distribution of the different types of violence. The map was generated with data from Natural Earth (http://www.naturalearthdata.com/) using the R package rnaturalearth v1.0.1 (https://github.com/ropensci/rnaturalearth) [30].

The spatial distribution of violence revealed notable geographical disparities in terms of prevalence levels and types of violence (Fig 4). When considering any form of violence, the first striking fact was that prevalence levels were high throughout the country (prevalence > 50%). However, certain regions stood out, such as Dakar in the first instance, followed by Tambacounda and Kédougou in the south-east of the country. The regions of Thiès, Louga and Saint-Louis in the north-west of the country had the lowest relative prevalence. The central, eastern and southern regions had an intermediate level of prevalence, giving the impression of a north-south continuum, but still remaining at a high level.

Psychological violence was more prevalent in the Dakar and Kédougou regions, while regions in the northern part of the country (Saint-Louis, Thiès, Louga, Matam) seemed less prone to this type of violence. Dakar still stood out in terms of physical violence, followed by the Tambacounda region. The rest of the country was characterized by a lower level of prevalence and less contrasting differences, with a slight contrast between the western and eastern parts of the country (Matam, Kaffrine, Sédhiou and Kédougou regions).

Domestic violence was more prevalent in eastern Senegal, particularly in the Tambacounda region, then southwards in Casamance (Sédhiou, Kolda, Kédougou regions) and Dakar. The regions of Ziguinchor, and then Matam and Louga northwards, had a lower level of prevalence. Inland regions (Fatick, Thiès, Diourbel, Kaffrine) had a prevalence of less than 25%.

Sexual violence seemed to divide the country into two parts: it was more frequently reported in the southern part of Senegal (Ziguinchor, Sédhiou, Kolda, Tambacounda and Kédougou regions), while it was less common in the northern part of the country. Neglect was concentrated in the central and northern regions (Dakar, Kaolack, Kaffrine, Louga, Diourbel and Saint-Louis).

#### Factors associated with violence.

The study examined socio-demographic characteristics associated with the experience of any form of violence, as well as with the experience of the most prevalent individual forms of violence, including psychological, verbal, physical, domestic, neglect-related and sexual violence (Table 3).

**Table 3:** Individual socio-demographic factors associated with different forms of violence. Odds ratios are presented for associations with p-values reaching statistical significance.

	Individual socio-demographic characteristics	N	Experienced violence n; % [95%CI]	No violence experienced n; % [95%CI]	p-value^1^ and OR [95%CI]
**ANY FORM OF VIOLENCE**	Gender				0.97
	Men	1,072	566; 52.80% [49.76% - 55.82%]	506; 47.20% [44.18% - 50.24%]	
	Women	1,102	581; 52.72% [49.72% - 55.70%]	521; 47.28% [44.30% - 50.28%]	
	Age				0.003
	5-18 years old	730	348; 47.67% [44.00% - 51.37%]	382; 52.33% [48.63% - 56.00%]	Reference
	19-59 years old	1,086	600; 55.25% [52.23% - 58.23%]	486; 44.75% [41.77% - 47.77%]	1.36 [1.12 - 1.64]
	60 + years old	358	199; 55.59% [50.27% - 60.78%]	159; 44.41% [39.22% - 49.73%]	1.37 [1.07 - 1.77]
	Marital status				<0.001
	Married	962	539; 56.03% [52.82% - 59.19%]	423; 43.97% [40.81% - 47.18%]	1.72 [1.35 - 2.19]
	Single	848	453; 53.42% [50.00% - 56.81%]	395; 46.58% [43.19% - 50.00%]	1.55 [1.21 - 1.98]
	Other	364	155; 42.58% [37.47% - 47.85%]	209; 57.42% [52.15% - 62.53%]	Reference
	Instruction				0.67
	Yes	1,601	849; 53.03% [50.55% - 55.49%]	752; 46.97% [44.51% - 49.45%]	
	No	573	298; 52.01% [47.83% - 56.16%]	275; 47.99% [43.84% - 52.17%]	
	Professional sector				0.25
	Public	92	48; 52.17% [41.56% - 62.60%]	44; 47.83% [37.40% - 58.44%]	
	Formal private	489	282; 57.67% [53.14% - 62.07%]	207; 42.33% [37.93% - 46.86%]	
	Informal private	64	42; 65.63% [52.61% - 76.75%]	22; 34.38% [23.25% - 47.39%]	
	Monthly salary (in XOF)				0.36
	<100k	398	237; 59.55% [54.53% - 64.38%]	161; 40.45% [35.62% - 45.47%]	
	100k-200k	184	98; 53.26% [45.79% - 60.59%]	86; 46.74% [39.41% - 54.21%]	
	≥200k	63	37; 58.73% [45.64% - 70.76%]	26; 41.27% [29.24% - 54.36%]	
**PSYCHOLOGICAL VIOLENCE**	Gender				0.40
	Men	1,072	498; 46.46% [43.44% - 49.49%]	574; 53.54% [50.51% - 56.56%]	
	Women	1,102	532; 48.28% [45.29% - 51.27%]	570; 51.72% [48.73% - 54.71%]	
	Age				<0.001
	5-18 years old	730	286; 39.18% [35.64% - 42.84%]	444; 60.82% [57.16% - 64.36%]	Reference
	19-59 years old	1,086	556; 51.20% [48.18% - 54.21%]	530; 48.80% [45.79% - 51.82%]	1.63 [1.35 - 1.97]
	60 + years old	358	188; 52.51% [47.20% - 57.77%]	170; 47.49% [42.23% - 52.80%]	1.72 [1.33 - 2.22]
	Marital status				<0.001
	Married	962	507; 52.70% [49.49% - 55.89%]	455; 47.30% [44.11% - 50.51%]	2.64 [2.05 - 3.43]
	Single	848	415; 48.94% [45.53% - 52.36%]	433; 51.06% [47.64% - 54.47%]	2.27 [1.75 - 2.96]
	Other	364	108; 29.67% [25.08% - 34.70%]	256; 70.33% [65.30% - 74.92%]	Reference
	Instruction				0.30
	Yes	1,601	748; 46.72% [44.26% - 49.20%]	853; 53.28% [50.80% - 55.74%]	
	No	573	282; 49.21% [45.05% - 53.39%]	291; 50.79% [46.61% - 54.95%]	
	Professional sector				0.30
	Public	92	46; 50.00% [39.99% - 60.01%]	46; 50.00% [39.99% - 60.01%]	
	Formal private	489	264; 53.99% [49.45% - 58.46%]	225; 46.01% [41.54% - 50.55%]	
	Informal private	64	40; 62.50% [49.47% - 74.02%]	24; 37.50% [25.98% - 50.53%]	
	Monthly salary (in XOF)				0.37
	<100k	398	224; 56.28% [51.25% - 61.19%]	174; 43.72% [38.81% - 48.75%]	
	100k-200k	184	92; 50.00% [42.85% - 57.15%]	92; 50.00% [42.85% - 57.15%]	
	≥200k	63	34; 53.97% [41.03% - 66.42%]	29; 46.03% [33.58% - 58.97%]	
**VERBAL VIOLENCE**	Gender				0.19
	Men	1,072	451; 42.07% [39.10% - 45.10%]	621; 57.93% [54.90% - 60.90%]	
	Women	1,102	494; 44.83% [41.87% - 47.82%]	608; 55.17% [52.18% - 58.13%]	
	Age				<0.001
	5-18 years old	730	256; 35.07% [31.63% - 38.67%]	474; 64.93% [61.33% - 68.37%]	Reference
	19-59 years old	1,086	515; 47.42% [44.42% - 50.44%]	571; 52.58% [49.56% - 55.58%]	1.67 [1.38 - 2.03]
	60 + years old	358	174; 48.60% [43.33% - 53.91%]	184; 51.40% [46.09% - 56.67%]	1.75 [1.35 - 2.26]
	Marital status				<0.001
	Married	962	467; 48.54% [45.35% - 51.75%]	495; 51.46% [48.25% - 54.65%]	2.63 [2.03 - 3.45]
	Single	848	382; 45.05% [41.67% - 48.47%]	466; 54.95% [51.53% - 58.33%]	2.29 [1.75 - 3.01]
	Other	364	96; 26.37% [21.98% - 31.27%]	268; 73.63% [68.73% - 78.02%]	Reference
	Instruction				0.63
	Yes	1,601	691; 43.16% [40.72% - 45.63%]	910; 56.84% [54.37% - 59.28%]	
	No	573	254; 44.33% [40.23% - 48.51%]	319; 55.67% [51.49% - 59.77%]	
	Professional sector				0.16
	Public	92	42; 45.65% [35.34% - 56.33%]	50; 54.35% [43.67% - 64.66%]	
	Formal private	489	247; 50.51% [45.99% - 55.02%]	242; 49.49% [44.98% - 54.01%]	
	Informal private	64	39; 60.94% [47.92% - 72.64%]	25; 39.06% [27.36% - 52.08%]	
	Monthly salary (in XOF)				0.20
	<100k	398	213; 53.52% [48.48% - 58.48%]	185; 46.48% [41.52% - 51.52%]	
	100k-200k	184	84; 45.65% [38.35% - 53.13%]	100; 54.35% [46.87% - 61.65%]	
	≥200k	63	31; 49.21% [36.52% - 61.99%]	32; 50.79% [38.01% - 63.48%]	
**PHYSICAL VIOLENCE**	Gender				0.28
	Men	1,072	364; 33.96% [31.14% - 36.89%]	708; 66.04% [63.11% - 68.86%]	
	Women	1,102	350; 31.76% [29.04% - 34.61%]	752; 68.24% [65.39% - 70.96%]	
	Age				0.073
	5-18 years old	730	262; 35.89% [32.43% - 39.51%]	468; 64.11% [60.49% - 67.57%]	
	19-59 years old	1,086	346; 31.86% [29.11% - 34.74%]	740; 68.14% [65.26% - 70.89%]	
	60 + years old	358	106; 29.61% [24.98% - 34.68%]	252; 70.39% [65.32% - 75.02%]	
	Marital status				0.15
	Married	962	295; 30.67% [27.78% - 33.70%]	667; 69.33% [66.30% - 72.22%]	
	Single	848	296; 34.91% [31.71% - 38.24%]	552; 65.09% [61.76% - 68.29%]	
	Other	364	123; 33.79% [28.99% - 38.94%]	241; 66.21% [61.06% - 71.01%]	
	Instruction				<0.001
	Yes	1,601	558; 34.85% [32.53% - 37.25%]	1,043; 65.15% [62.75% - 67.47%]	Reference
	No	573	156; 27.23% [23.66% - 31.10%]	417; 72.77% [68.90% - 76.34%]	0.70 [0.57 - 0.86]
	Professional sector				<0.001
	Public	92	22; 23.91% [15.90% - 34.15%]	70; 76.09% [65.85% - 84.10%]	Reference
	Formal private	489	157; 32.11% [28.02% - 36.48%]	332; 67.89% [63.52% - 71.98%]	1.50 [0.91 - 2.57]
	Informal private	64	34; 53.13% [40.33% - 65.55%]	30; 46.88% [34.45% - 59.67%]	3.61 [1.83 - 7.25]
	Monthly salary (in XOF)				0.65
	<100k	398	128; 32.16% [27.64% - 37.03%]	270; 67.84% [62.97% - 72.36%]	
	100k-200k	184	61; 33.15% [26.50% - 40.52%]	123; 66.85% [59.48% - 73.50%]	
	≥200k	63	24; 38.10% [26.41% - 51.23%]	39; 61.90% [48.77% - 73.59%]	
**DOMESTIC VIOLENCE**	Gender				0.016
	Men	1,072	184; 17.16% [14.98% - 19.59%]	888; 82.84% [80.41% - 85.02%]	Reference
	Women	1,102	148; 13.43% [11.50% - 15.62%]	954; 86.57% [84.38% - 88.50%]	0.75 [0.59 - 0.95]
	Age				0.23
	5-18 years old	730	125; 17.12% [14.50% - 20.10%]	605; 82.88% [79.90% - 85.50%]	
	19-59 years old	1,086	157; 14.46% [12.45% - 16.72%]	929; 85.54% [83.28% - 87.55%]	
	60 + years old	358	50; 13.97% [10.63% - 18.09%]	308; 86.03% [81.91% - 89.37%]	
	Marital status				0.31
	Married	962	137; 14.24% [12.13% - 16.65%]	825; 85.76% [83.35% - 87.87%]	
	Single	848	142; 16.75% [14.33% - 19.47%]	706; 83.25% [80.53% - 85.67%]	
	Other	364	53; 14.56% [11.19% - 18.70%]	311; 85.44% [81.30% - 88.81%]	
	Instruction				0.25
	Yes	1,601	253; 15.80% [14.07% - 17.70%]	1,348; 84.20% [82.30% - 85.93%]	
	No	573	79; 13.79% [11.13% - 16.95%]	494; 86.21% [83.05% - 88.87%]	
	Professional sector				0.22
	Public	92	12; 13.04% [7.21% - 22.06%]	80; 86.96% [77.94% - 92.79%]	
	Formal private	489	80; 16.36% [13.25% - 20.01%]	409; 83.64% [79.99% - 86.75%]	
	Informal private	64	15; 23.44% [14.12% - 35.98%]	49; 76.56% [64.02% - 85.88%]	
	Monthly salary (in XOF)				0.57
	<100k	398	70; 17.59% [14.05% - 21.77%]	328; 82.41% [78.23% - 85.95%]	
	100k-200k	184	26; 14.13% [9.60% - 20.20%]	158; 85.87% [79.80% - 90.40%]	
	≥200k	63	11; 17.46% [9.45% - 29.52%]	52; 82.54% [70.48% - 90.55%]	
**NEGLECT**	Gender				0.50
	Men	1,072	96; 8.96% [7.35% - 10.86%]	976; 91.04% [89.14% - 92.65%]	
	Women	1,102	108; 9.80% [8.14% - 11.75%]	994; 90.20% [88.25% - 91.86%]	
	Age				<0.001
	5-18 years old	730	38; 5.21% [3.76% - 7.14%]	692; 94.79% [92.86% - 96.24%]	Reference
	19-59 years old	1,086	129; 11.88% [10.04% - 13.99%]	957; 88.12% [86.01% - 89.96%]	2.45 [1.70 - 3.61]
	60 + years old	358	37; 10.34% [7.47% - 14.08%]	321; 89.66% [85.92% - 92.53%]	2.10 [1.31 - 3.37]
	Marital status				<0.001
	Married	962	113; 11.75% [9.81% - 13.99%]	849; 88.25% [86.01% - 90.19%]	4.27 [2.38 - 8.50]
	Single	848	80; 9.43% [7.59% - 11.65%]	768; 90.57% [88.35% - 92.41%]	3.34 [1.83 - 6.72]
	Other	364	11; 3.02% [1.60% - 5.50%]	353; 96.98% [94.50% - 98.40%]	Reference
	Instruction				0.30
	Yes	1,601	144; 8.99% [7.66% - 10.53%]	1,457; 91.01% [89.47% - 92.34%]	
	No	573	60; 10.47% [8.14% - 13.34%]	513; 89.53% [86.66% - 91.86%]	
	Professional sector				0.28
	Public	92	8; 8.70% [4.10% - 16.90%]	84; 91.30% [83.10% - 95.90%]	
	Formal private	489	59; 12.07% [9.38% - 15.36%]	430; 87.93% [84.64% - 90.62%]	
	Informal private	64	11; 17.19% [9.29% - 29.10%]	53; 82.81% [70.90% - 90.71%]	
	Monthly salary (in XOF)				0.96
	<100k	398	47; 11.81% [8.89% - 15.49%]	351; 88.19% [84.51% - 91.11%]	
	100k-200k	184	23; 12.50% [8.25% - 18.37%]	161; 87.50% [81.63% - 91.75%]	
	≥200k	63	8; 12.70% [6.03% - 24.04%]	55; 87.30% [75.96% - 93.97%]	
**SEXUAL VIOLENCE**	Gender				0.014
	Men	1,072	30; 2.80% [1.93% - 4.02%]	1,042; 97.20% [95.98% - 98.07%]	Reference
	Women	1,102	53; 4.81% [3.66% - 6.29%]	1,049; 95.19% [93.71% - 96.34%]	1.75 [1.12 - 2.80]
	Age				0.047
	5-18 years old	730	23; 3.15% [2.05% - 4.76%]	707; 96.85% [95.24% - 97.95%]	Reference
	19-59 years old	1,086	52; 4.79% [3.63% - 6.28%]	1,034; 95.21% [93.72% - 96.37%]	1.55 [0.95 - 2.59]
	60 + years old	358	8; 2.23% [1.04% - 4.53%]	350; 97.77% [95.47% - 98.96%]	0.70 [0.29 - 1.52]
	Marital status				0.054
	Married	962	39; 4.05% [2.94% - 5.55%]	923; 95.95% [94.45% - 97.06%]	2.52 [1.14 - 6.68]
	Single	848	38; 4.48% [3.23% - 6.16%]	810; 95.52% [93.84% - 96.77%]	2.80 [1.26 - 7.42]
	Other	364	6; 1.65% [0.67% - 3.73%]	358; 98.35% [96.27% - 99.33%]	Reference
	Instruction				0.22
	Yes	1,601	66; 4.12% [3.23% - 5.25%]	1,535; 95.88% [94.75% - 96.77%]	
	No	573	17; 2.97% [1.79% - 4.81%]	556; 97.03% [95.19% - 98.21%]	
	Professional sector				0.37
	Public	92	2; 2.17% [0.38% - 8.38%]	90; 97.83% [91.62% - 99.62%]	
	Formal private	489	26; 5.32% [3.57% - 7.80%]	463; 94.68% [92.20% - 96.43%]	
	Informal private	64	4; 6.25% [2.02% - 16.02%]	60; 93.75% [83.98% - 97.98%]	
	Monthly salary (in XOF)				0.26
	<100k	398	24; 6.03% [3.98% - 8.96%]	374; 93.97% [91.04% - 96.02%]	
	100k-200k	184	7; 3.80% [1.68% - 7.99%]	177; 96.20% [92.01% - 98.32%]	
	≥200k	63	1; 1.59% [0.08% - 9.68%]	62; 98.41% [90.32% - 99.92%]	

^1^Pearson’s Chi-squared test. Abbreviation: CI = Confidence Interval. OR = Odds Ratio.

Both age (p = 0.003) and marital status (p < 0.001) were significantly associated with the experience of any form of violence. Elderly individuals (OR=1.37, 95% CI: 1.12–1.64) and adults (OR=1.36, 95% CI: 1.07–1.77) were more likely than children and adolescents to have experienced violence. Married (OR=1.72, 95% CI: 1.35–2.19) and single individuals (OR=1.55, 95% CI: 1.21–1.98) were more likely than those with other marital statuses to have experienced violence.

When examining specific forms of violence, age and marital status remained significantly associated with psychological violence, verbal, and neglect-related violence, while distinct patterns emerged for physical, domestic and sexual violence.

Elderly individuals were more likely than children and adolescents to have experienced psychological (OR=1.72, 95% CI: 1.33–2.22), verbal (OR=1.63, 95% CI: 1.35–1.97) or neglect-related (OR=2.10, 95% CI: 1.31–3.37) violence, with similar findings for adults (OR=1.63, 95% CI: 1.35–1.97; OR=1.67, 95% CI: 1.38–2.03; and OR=2.45, 95% CI: 1.70–3.61, respectively). Married individuals were more likely than those with other marital statuses to have experienced psychological (OR=2.64, 95% CI: 2.05–3.43), verbal (OR=2.63, 95% CI: 2.03–3.45) or neglect-related (OR=4.27, 95% CI: 2.38–8.50) violence, with similar findings for single individuals (OR=2.27, 95% CI: 1.75–2.96; OR=2.29, 95% CI: 1.75–3.01; and OR=3.34, 95% CI: 1.83–6.72, respectively).

Gender was the only factor significantly associated with domestic violence (p = 0.016). Men were more likely than women to experience this form of violence (OR=1.34, 95% CI: 1.06–1.69).

Both gender (p = 0.014) and age (p = 0.047) were significantly associated with sexual violence, while marital status showed a borderline association (p = 0.053). Women were more likely than men to experience sexual violence (OR=1.75, 95% CI: 1.12–2.80). Married (OR=2.52, 95% CI: 1.14–6.68) and single individuals (OR=2.80, 95% CI: 1.26–7.42) were more likely than those with other marital statuses to have experienced sexual violence. Age-related differences were not conclusive.

Both instruction (p < 0.001) and professional sector (p < 0.001) were significantly associated with physical violence. Individuals working in the informal private sector were more likely than those in the public sector to experience physical violence (OR=3.61, 95% CI: 1.83–7.25), while no statistically significant difference was observed for those working in the formal private sector (OR=1.50, 95% CI: 0.91–2.57). Individuals with no instruction were less likely than those with instruction to have experienced physical violence (OR=0.70, 95% CI: 0.57–0.86).

#### Assistance-seeking behaviour.

The study revealed that victims of violence rarely seek any form of assistance (Table 4). Among those who reported having experienced violence, nine out of ten (89.53%) reported never having alerted the police, eight out of ten (79.46%) had not sought medical assistance, and more than nine out of ten (93.8%) had not sought psychological support following such events. Women were even less likely than men to seek assistance.

**Table 4:** Police, medical and psychological assistance in cases of violence, by gender.

Features	Violence (any form) N = 1,147	Male N = 566	Female N = 581	p-value
Police alert				0.90
Never	231 (89.53%)	128 (87.67%)	103 (91.96%)	
Sometimes	4 (1.55%)	3 (2.05%)	1 (0.89%)	
Rarely	14 (5.43%)	9 (6.16%)	5 (4.46%)	
Often	3 (1.16%)	2 (1.37%)	1 (0.89%)	
Always	6 (2.33%)	4 (2.74%)	2 (1.79%)	
Healthcare support				0.20
Never	205 (79.46%)	111 (76.03%)	94 (83.93%)	
Sometimes	11 (4.26%)	8 (5.48%)	3 (2.68%)	
Rarely	36 (13.95%)	21 (14.38%)	15 (13.39%)	
Often	5 (1.94%)	5 (3.42%)	0 (0.00%)	
Always	1 (0.39%)	1 (0.68%)	0 (0.00%)	
Psychological support				0.69
Never	242 (93.80%)	136 (93.15%)	106 (94.64%)	
Sometimes	2 (0.78%)	2 (1.37%)	0 (0.00%)	
Rarely	13 (5.04%)	7 (4.79%)	6 (5.36%)	
Often	1 (0.39%)	1 (0.68%)	0 (0.00%)	
Always	0 (0.00%)	1 (0.00%)	0 (0.00%)	

### 1.9. Suicide risk

**Fig 5:**
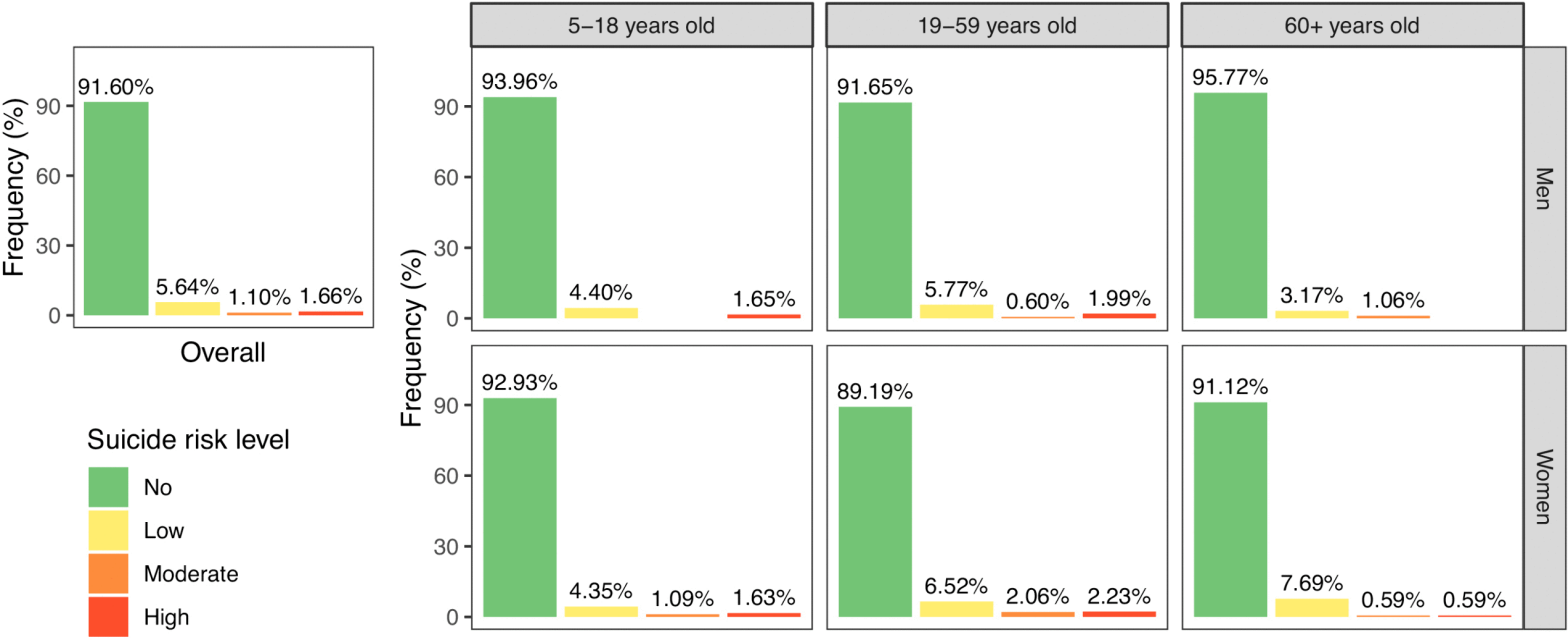
Distribution of suicide risk levels by age and gender. Suicide risk levels are coded by colour: green for no risk, yellow for low risk, orange for moderate risk, and red for high risk.

Overall, 8.40% of respondents exhibited some level of suicide risk (Fig 5). Individuals at high risk accounted for 1.66% of the sample and were more prevalent than those at moderate risk (1.10%).

#### Prevalence of suicide risk by age and gender.

Gender- and age- disaggregated analysis showed that overall women had a higher prevalence of suicide risk compared to men, and adults aged 19–59 years were more affected than other age categories (Fig 5). Women aged 19–59 emerged as the most vulnerable subgroup, with the highest rates of both high (2.23%) and moderate (2.06%) suicide risk. Among men, those aged 19–59 had the highest prevalence of high suicide risk (1.99%). Among youth aged 5–18, early signs of suicide risk were observed across all categories, with no gender difference for low and high risk. In contrast, elderly individuals aged 60 and older showed a lower prevalence of high suicide risk compared to the survey population average. Moderate risk in this age group was either lower or similar to the population average, while women’s low suicide risk was notably higher (7.69%).

#### Suicide risk and neuropsychiatric disorders.

**Fig 6:**
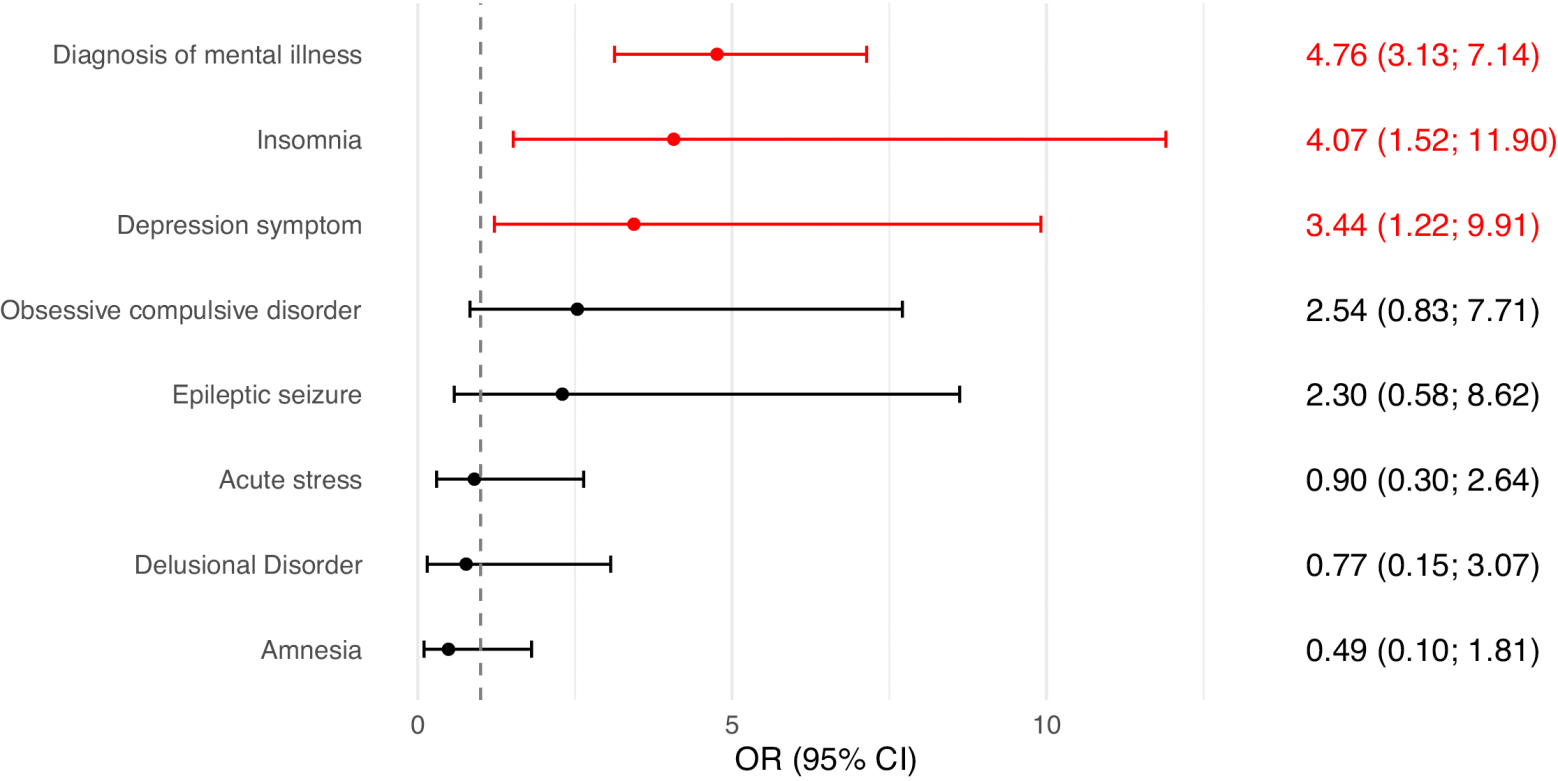
Association between neuropsychiatric disorders and suicide risk. Abbreviations: CI = Confidence Interval; OR = Odds Ratio.

The diagnosis of a mental disorder emerged as the factor most strongly associated with suicide risk, with an OR of 4.76 (95% CI: 3.13–7.14), indicating that affected individuals are nearly five times more likely to exhibit suicidal behaviour compared to those without such a diagnosis (Fig 6). Insomnia also showed a significant association, with an OR of 4.07 (95% CI: 1.52–11.90), suggesting a strong link between sleep disturbances and psychological vulnerability. Depressive symptoms were associated with an OR of 3.44 (95% CI: 1.22–9.91). In contrast, other conditions such as obsessive-compulsive disorder, epileptic seizures, acute stress, delusional disorders, and amnesia did not show statistically significant associations with suicide risk.

#### Suicide risk and violence.

**Fig 7:**
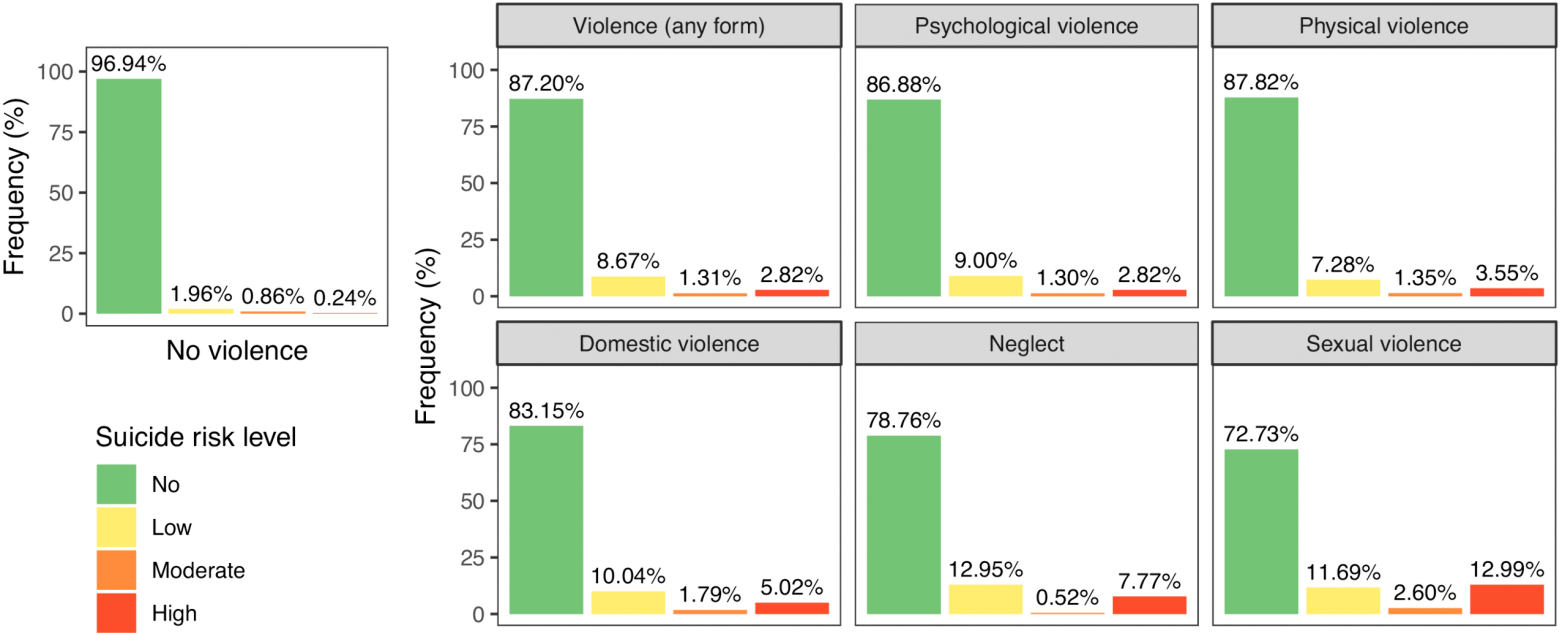
Distribution of suicide risk levels by form of violence. Suicide risk levels are coded by colour: green for no risk, yellow for low risk, orange for moderate risk, and red for high risk.

Among individuals not exposed to violence, the overall prevalence of suicide risk was 3.06% (Fig 7), with a descending gradient from low (1.96%) to moderate (0.86%) and high risk (0.24%). In contrast, those exposed to any form of violence exhibited a prevalence over four times higher, at 12.80%. In this population, while low risk remained the most common (8.67%), high risk (2.82%) exceeded moderate risk (1.30%), accounting for approximately 22% of all individuals identified as at risk. Elevated rates of high suicide risk were observed across all forms of violence, though the prevalence varied by form. It was the highest among individuals exposed to sexual violence, where an alarming 12.99% were classified as high risk (representing 47.6% of all at-risk individuals in this population), followed by neglect (7.77%) domestic violence (5.02%), and physical violence (3.55%). Although psychological violence was associated with the lowest high-risk rate (2.82%) among all forms of violence, this rate was still more than ten times higher than the one observed in the population not exposed to violence.

## Discussion

This study presents the first comprehensive analysis of violence patterns and their association with suicide risk in Senegal, contributing to address the knowledge gap in population mental health in Sub-Saharan Africa. The findings offer a valuable evidence base to inform the development of a national public health action plan in Senegal for preventing violence and suicide, with targeted interventions for vulnerable populations [34]. In the remainder of this discussion, we briefly compare our results with epidemiological findings from other African settings and explore attitudinal, behavioural, socio-cultural, economic, and political factors that may contribute to the high prevalence of violence and suicide risk in Senegal. Using a programmatic lens, we aim to better highlight key drivers and identify actionable priorities, including awareness-raising efforts and the development of comprehensive care services for victims of violence.

### 1.10. Social norms inhibiting violence disclosure

The study showed that violence is both widespread and multifaceted in Senegal, with emotional and behavioural forms of abuse more prevalent than physical harm. These findings are consistent with systematic reviews documenting high rates of violence and polyvictimization among youths in other African and LMIC settings [35,36], although lower than those reported in South Africa [37]. This raises important questions about the level of societal tolerance for different forms of violence in Senegal, where many face structural fragility due to youth demographics, socio-economic hardship, gender inequality and precarious urban living conditions. Cultural and social norms may inhibit the open expression of emotions and experience related to violence. Attitudes and discourse are generally governed by different values that do not encourage the open expression of emotions and affects. During interpersonal exchanges, it is frowned upon or considered impolite to broach subjects considered socially sensitive, and to cause embarrassment to one’s interlocutor. Discretion or modesty (*kersa* in Wolof) implies moderating, euphemizing or even minimizing an expression felt to be socially inappropriate or disturbing. Avoidance of topics considered complicated to raise within the family or social circle can lead to complete silence (*nopi* in Wolof). Individuals may censor themselves rather than risk rejection or reprimand, being compelled not to raise the subject again, for fear of being discredited, mocked or excluded. This form of self-censorship and repression of affects, rooted in strong social control, has consequences in terms of mental health (depression, for example) and violence [20]. Violence is not seen as a matter for public, legal, social or health action, but as a problem to be solved and contained within the intimate family sphere. In addition to the anthropological rules governing speech and social relations, there is the habitus of *moung, a* Wolof concept that refers to the obligation to accept one’s fate and bend to destiny. This notion is expressed here in Wolof, as it is the vernacular language of the country, particularly in an urban context. However, in a context of multilingualism and the persistence of ethnic identities in Senegal, individuals alternate between languages according to social contexts and interlocutors. Similar vision of individual destiny can be found in other ethnic groups. Among the Halpulaar, for example, the notion of *pulakuu* refers to the central value of modesty. When a woman complains about the challenges she faces in her marital life or in her relationship with her mother-in-law, about the suffering and misfortune she is going through, those around her will tell her that she must be patient, endure and accept. Individuals are educated to accept the primacy of the social order and to submit to collective interests over the personal expression of their subjectivity. Thus, rather than denouncing physical or sexual violence within the family, social pressure pushes family members to defend the honour of elders and the family’s reputation in the community by preserving appearances, whatever the cost may be for some of them. Wolof upbringing fosters the internalization by children of a habitus that includes respect for authority, the quest for respectability, obedience, and a good understanding of relationships within kinship and the social sphere [38]. The under-reporting of violence is also linked to the social status of those involved in the social or family relationship leading to the violence. Gender-based violence is most often addressed within the context of family and close relations, as Senegalese society has a tradition of resorting to mediation in the face of certain socially sensitive problems [39]. The aim of customary mediation is to find a solution acceptable to all parties, while containing the scandal or moral offence. Its purpose is not to offer comfort to the victim, nor to provide legal or psychosocial assistance, but rather to stifle a situation deemed embarrassing and humiliating if, by misfortune, it were to become public within the community. As a result, teenage girls or young women who have been raped or sexually assaulted within the extended family or community are forced to marry their attacker. The characterization of rape is then dissolved within the marriage - which is civilly and religiously legitimized - redefining the relationship between the spouses and preventing the expression of complaints. In this way, the honourability of both the perpetrator’s and victim’s families is preserved, while the victim is left to deal with her traumas alone, in addition to denying her suffering. In some cases, socially disturbing violence (rape, sexual violence) is erased by a form of social regulation (forced marriage, early marriage) with violent and lasting repercussions (rape within marriage, submission to an unfair decision, unwanted pregnancies, dropping out of school). A patriarchal vision of masculinity (valuing robustness, risk-taking and the defence of honour) and loopholes in law enforcement mechanisms contribute to the social dynamics that foster the superimposition of different forms of violence [40]. The disclosure of violence is also modulated by the status of individuals and economic resources within families. In a study on elder violence [41] economic insecurity is a risk factor for violence in a society where “*medical care is a divisive factor in many families*” [42]. The likelihood of violence is heightened by the absence of alternatives to family solidarity in a demographic context of aging. Older people tend to downplay and try to excuse the violence they suffer at the hands of their relatives. Counterintuitively, given the representations associated with the moral economy of the family in West Africa, this denial is even stronger because it refers to a little-known reality – elder violence in a context of ageing – and runs counter to an African imaginary that is supposed to uphold respect for elders and intra-family solidarity [41]. Although the elderly are not the category most exposed to violence, they are not “*immune*” to it simply because of their age.

### 1.11. Violence patterns in a society in transition

A first hypothesis for the observed spatial gradient relates to the cultural heterogeneity and distribution of ethnic groups in Senegal. According to observers, people in the south of the country are more likely to report violent attitudes or practices, while social groups in the north are more reserved, viewing these behaviours as a private matter for cultural or religious reasons that need to be explored in greater depth. Furthermore, given the recent socio-political context (social protest, political demonstrations linked to the electoral process, student demands, industrial strikes), certain regions of the country, such as Dakar and Casamance in the south, have experienced more violent events in their popular expressions and in the violent repression exercised by the authorities (arrests, injuries and deaths of demonstrators following police shootings) in March 2021 [43] and June 2023 [44]. Such proximity to violence may contribute to normalizing some of its manifestations and making them more socially expressible, particularly physical and psychological violence. In Casamance, the region’s forty-year struggle for independence, and the conflicts between independence fighters and the central government, lend historical depth to this relationship with violence. An estimated 60,000 people have been displaced and 5,000 have died in this so-called “low-intensity” conflict, and despite a period of calm, the region remains in a state of crisis. Events in Casamance are attracting considerable national and regional attention because of their impact on the country’s social and political stability. They were triggered by multifactorial factors, including political demands, social tensions and the economic crisis. The complexity of the factors underlying the protests and the ensuing violence calls for an in-depth and nuanced analysis, taking into account the political, social and economic dimensions of the conflict, as well as the subjective perspectives of the various stakeholders. Violence due to neglect, while not reaching the levels of other types of violence, occurs more frequently in the centre and north of the country. These regions (Dakar, Kaolack, Kaffrine, Louga, Diourbel and Saint-Louis) constitute the area of influence of the *daaras*, where many children (mainly boys) have been placed by their parents to receive Koranic training. In Senegal, the question of talibé children from *daaras* is a thorny political and social issue. Irrespective of assessments of the quality of teaching and pedagogical practices, the harsh living conditions imposed on *taalibe* (financial and nutritional begging, ill-treatment, forced labour, exploitation, estrangement from family) raise questions, even if this educational model retains a certain appeal [45]. Child entrustment, defined as “the delegation of parental roles to persons other than the biological parents”, is a common practice in West Africa. Traditionally, the effects of separation are absorbed by community life, extended kinship and the identification of substitute parents. Nevertheless, society recognises the psychic cost of the separation between children and parents, since the Wolof notion of *guélou* designates the suffering associated with the break-up, which is also ritualized [46]. This type of upbringing, *yaar doom*, implies the abdication of parental responsibilities, as parents feel confident in their kin or in the institution in which they place their child, and do not feel entitled to check on the child’s living conditions and development, thus exposing him or her to violence and failure to respect his or her rights. Some expressions show the endorsement of “education by the stick” (*yaar*), as well as the lack of control and abandonment of the child (*yakh rek lala ladj*: just give me back his bones). Children in care do not always receive the attention and care they need, leading to psychological distress. This passive violence can escalate into active violence (physical, verbal and sexual violence) by adults if the child rebels against his or her situation. Children then experience a stressful situation, perceiving themselves as unworthy of love, and seeing those around them as unavailable or rejecting. They develop neurodevelopmental disorders. The socio-economic logics (regulation of social inequalities, demographic adjustment, reinforcement of solidarities) behind fostering have emotional, psychological and relational consequences for these children. The high levels of psychological and physical violence across all regions, and to a lesser extent those of domestic violence, reveal traditional forms of social regulation framed by strong relationships of authority, which can be associated with the expression of various forms of violence. The “*principles that govern the authority of the father of the family, the husband, the marabouts are considered universal and transcendent, which makes these social relationships unquestionable and uncontested*” [47]. In this perspective, psychologists highlight the problems and suffering associated with the transition to modernity and “the changes that are redefining intra-family ties and a new parenthood” [48]. The context of economic crisis (inflation, difficulties experienced by young people in accessing the training and jobs they desire) since the Covid-19 pandemic in 2020–2021 contributes to exacerbating family and social tensions. Mining regions are characterized by difficult working and living conditions, trafficking, prostitution and exploitation of certain social categories (e.g., minors, women, migrants), all of which are associated with violent behaviour. Diffuse social dissatisfaction and reduced well-being are also expressed through the desire to emigrate to the European Union or North America, in particular [49], despite the morbid risks associated with irregular migration routes [50]. Finally, everyday living conditions also fuel expressions of violence, particularly in urban areas. Dakar constitutes a specific context: from a socio-demographic point of view, the country’s capital concentrates one-fifth of the population and remains an attractive region for the country’s various social groups. The very high densities [22] contribute to increased promiscuity within households and tensions between neighbours. Traffic is often chaotic (traffic jams, non-compliance with traffic regulations, state of means of transport), resulting in wasted time, fatigue and frustration. People carry out their daily and professional activities in a constant state of stress. However, living conditions in the Dakar region vary widely between the most privileged neighbourhoods and the outlying suburbs, and would benefit from a spatial analysis on a finer scale. Pollution further undermines mental health, leaving Dakar’s inhabitants, who are emotionally and physically weakened, more prone to violent (physical and verbal) behaviour.

### 1.12. Gender dimensions of violence

We found that patterns of victimization differed by gender, consistent with global evidence that women are disproportionately affected by physical or sexual violence, or both, from an intimate partner in their lifetime [51]. In West Africa, sexual violence is also frequent, as highlighted by a recent study [13]. The reasons are complex and may be linked to unequal power structures within couple relationships and women’s status in society, although there may be variations according to ethnic and social groups. Despite prevailing gender inequalities and socio-cultural norms that privilege male dominance and reinforce the subordination of women, it is important to acknowledge that men can also be victims of violence, though patterns and rates of victimization differ. Our findings indicate that male victimization, particularly in cases of domestic and sexual violence, may be more prevalent than previously understood in Senegal. A recent scoping review underscored this gap, noting that male experiences of violence remain significantly understudied across various contexts and forms [52]. More specific to Senegal, in a retrospective study conducted in the Kolda region and based on court records of cases of sexual violence involving minor victims between 1992 and 2011, it was found that most victims were unmarried (96.9% of women and 72.2% of men). Victims of sexual violence knew their attackers in 66% of cases, and 54.9% of victims had been raped. Their average age was 12.3 years (±3 years), while that of the aggressors was 26.4 years (±9.5 years) [18]. The perception of marital and sexual violence is justified by mystical representations and gender domination. In most cases, the husband is not to blame for the violence, and many excuses are found to explain the violence suffered [53]. In the case of incestuous, alcoholic fathers, violence is explained by the intervention of dark magical forces. Some women report that it is normal for a wife to be corrected by her husband in the event of misconduct [54]. Conjugal violence, whether sexual or psychological, is part of a continuum of exposure to violence, of which the conjugal sphere is an integral part [55] and “*has to be interpreted within broader norms that create vulnerability to violence for women*” [56]. Women are exposed to violence in the various spheres (marital, professional, educational, etc.) in which they carry out their activities over the course of their biography. Certain factors (age gap between spouses, women’s youth, having a mental or physical handicap, material conditions, having suffered violence during childhood or adolescence, precarious social status or social isolation) constitute risks of experiencing domestic violence and put certain groups at greater risk, even if domestic violence affects all social backgrounds. This exposure to violence is linked to the configuration and pathways of unions.

### 1.13. Epidemiological and sociocultural dimensions of suicide in Senegal

This study identified a concerning minority of individuals at high risk of suicide, with elevated risk among (i) women 18–59 years old, (ii) individuals who have experienced violence, and (iii) individuals with mental illness. These findings confirm suicide is an important public health problem in Senegal, consistent with other African contexts [57]. The prevalence of suicide risk using MINI V5 was around 7% among children and adolescents aged 5–18 years, lower than the Global School-Based Student Health Survey estimates of over 20% for suicidal ideation or planning among the group of older adolescents aged 13–17 years in the African region [58,59]. Gender also plays a critical role, with adolescent girls consistently reporting higher rates of suicidality than boys, a pattern that aligns with both our data and regional evidence [58]. Rare suicide attempts among children have been described within in their family and religious contexts [60]. Such findings raise critical questions about how children’s suffering is recognized, interpreted, and responded to within families. Modernization and globalization are inducing two contradictory models of education within African families, particularly in urban areas [61]. Young people must simultaneously navigate expectations rooted in their culture of origin, while adapting to modern standards that are not always well defined and understood. This transition can induce distress, guilt, and identity conflict, all of which increase vulnerability to suicidal behaviour. In contrast, adults may be less likely to seek help for emotional distress (associated with depression and internalized symptoms), which can increase the risk of suicide. Importantly, while women are more likely to experience suicide attempts, their suicide rate is lower than that of men [62]. In Dakar, men die by suicide twice as often as women, with deaths concentrated among young adults (21–30 years) living in peri-urban areas, where professional, family, financial, and health-related stressors intersect [63]. Sociologists and social psychologists point to the social mutations affecting increasingly vulnerable individuals, particularly in urban settings, where traditional systems of protection are no longer effective. Individuals are no longer able to cope with family pressure and the multiple demands placed on them. When faced with perceived failure, the weight of shame can become overwhelming [47]. The role of shame in the traditional upbringing process is repeatedly emphasized [64], as it is used to help the individual maintain his role in the group, without which he will publicly signify his failure. The social scene is described as “*a theatre of submission and permanent repression*”, while psychologist Ferdinand Ezembé describes it as “*the kinship of fear*”. Elderly individuals face their own set of vulnerabilities, including social isolation, declining autonomy linked to their state of physical or mental health. The mechanisms that lead to suicide after bereavement are not fully analysed, but it is likely that feelings of shame, responsibility and stigma operate [65]. The more isolated and misunderstood individuals feel, the more vulnerable they become to suicide. Beyond age and gender differences, the study also contributed to the growing body of evidence highlighting a strong association between exposure to violence – particularly sexual violence – and poorer physical and mental health, including depression, anxiety and suicide risk [66–69]. Systematic reviews confirmed high prevalence of depression, anxiety, and suicidal behaviour among West African adolescents, with violence and trauma as major contributors [70]. Lifetime exposure to sexual violence has been shown to more than double the odds of poor mental health among women in sub-Saharan Africa [68]. The psychological consequences of violence are also gendered, with women often bearing a disproportionate burden even when the exposure is similar [66,67]. Our findings indicate that the risk of suicide among individuals with mental illness falls within the range previously reported in the literature, namely 3–12 times higher than in people without psychiatric illness [12]. The prevalence of psychotic disorders in Africa has been estimated to range from 1.0% to 4.4% [71]. Psychological manifestations (e.g., anxiety-depressive and psychosomatic disorders, delusions) are cries for help that are often not properly recognized. In particular, the lack of reliable social support for those with schizophrenia can leave them feeling alone and unwanted, continuing to suffer from the stigma attached to mental illness in their communities [72]. Taken together, all these findings underscore the urgent need for coordinated prevention strategies that consider both clinical and social dimensions, and that are sensitive to differences across age and gender.

### 1.14. Barriers to accessing post-violence support services

The consistently low levels of assistance-seeking behaviour observed in this study highlight substantial barriers to accessing post-violence services in Senegal, including legal, police, and medico-social services. One major barrier is that being subjected to violence seems to constitute a form of normalcy integrated into social functioning, since victims do not turn to the institutions supposed to embody a form of protection or care (medical, social, judicial). Explanations related to social organization, family and kin-based educational processes, and the taboo nature of certain types of violence, as discussed above, enable us to propose socio-anthropological explanations. In general, “such matters” are expected to be managed within a restricted circle, to avoid public exposure to shame. Victims are often dissuaded from denouncing violence perpetrators, who often interact daily with their victims in the home, school and community [73,74]. Whether perpetrators are parents or other guardians, peers, romantic partners or strangers, the victim is forced to remain silent, and suffers a double suffering, one real and the other symbolic. According to Bourdieu, symbolic violence is that coercion which is only instituted through the adherence that the dominated cannot fail to grant to the dominant - and therefore to domination - when, in order to think of him and of themselves, or better still, to think of their relationship with him, they only have instruments in common with him [75]. Victims remain in a cycle of guilt when the community holds them responsible for their fate. A second barrier is the lack of awareness about available services, not only among victims but also among their families and social circles [76]. Globally, the use of mental health services remains alarmingly low, particularly in LMICs, with large treatment gaps observed even for common conditions like anxiety and depression [77]. This is particularly acute in the West African context where mental health remains a marginalized public health issue suffering from chronic underinvestment [78]. In this region, mental health care services and those for adolescents are too scarce to meet the need for care [79] and the numbers of specialized professionals (psychiatrists, psychologists) falls far below the WHO-recommended standards [80]. Furthermore, mental health in Senegal is not integrated into primary health care, which encourages the stigmatization of people suffering from mental disorders. Mental health professionals in the country have highlighted the lack of structured, professionalized support services for victims, including safe spaces for psychological assistance and crisis response. A third barrier is the insufficient training of frontline professionals (including police officers, magistrates, doctors, psychiatrists, psychologists, educators, social workers, and firefighters) in the care and support of individuals affected by violence. Investigative procedures used when interviewing victims of violence can expose them to socio-religious prejudices regarding suicidal behaviour, moral representations associated with “good morals” and the stigmatization of mental illness [81], which can worsen their psychological experience and even increase the risk of suicide. In the case of sexual violence, the institutional framework does not appear to be conducive to hearing traumatized voices [82]. Addressing these barriers requires a coordinated, multi-sectoral response. Encouragingly, some progress has been made recently in Senegal. Psychosocial support for victims in the event of a crisis has only been integrated into Senegal’s Centre des Opérations d’Urgences Sanitaires (COUS) [83]. Additionally, Senegal has launched an awareness-raising campaign aimed at combating verbal and psychological violence, sexism and gender inequality, in line with the March 8 theme of consolidating the national commitment to combating violence against women and girls. As part of this campaign, the Italian Agency for Development Cooperation (AICS) has collaborated with the Senegalese Ministry for Women, the Family and Child Protection to set up “*Wallu Allo 116*”, a listening, guidance and information centre dedicated to gender-based violence. This free service, launched in November as part of the Support Project for the National Strategy for Gender Equity and Equality (PASNEEG II), operates 24/7 to provide protection and support for victims. After just one month of operation, the centre has already received more than 1,700 calls from the country’s 14 regions, demonstrating the scale of the violence and the urgent need of a reinforced institutional response [84].

### 1.15. Strengths, limitations and perspectives

A key strength of this study lies in its structured, population-based approach to assessing violence, in contract to the traditional unstructured methods which rely on what Hart refers to as the “*charismatic authority*” of the researcher [85] and where the credibility of an assessment is based on their specialized training, diplomas, experience or intuition. While these approaches may be valuable in clinical or qualitative settings, they are insufficient at the population level, where a rigorous epidemiological methods are needed to ensure the conceptual and statistical validity of violence assessment results. This study adhered to epidemiological standards while adapting its tools and procedures to the Senegalese socio-cultural context to maximize its plausibility. That said, several limitations warrant consideration. First, although the questionnaire was adapted from validated tools used in similar contexts [86], its psychometric properties were not formally assessed in the Senegalese setting. Second, it did not capture the temporality, severity or frequency of violence. As a result, it was not possible to distinguish between isolated incidents and chronic or severe abuse, nor to examine associations between specific types of violence and the prevalence of psychological disorders. Future research should incorporate these measures to provide a more comprehensive understanding of the impact of violence on mental health. Third, social desirability bias may have influenced responses, particularly around culturally sensitive topics such as sexual or domestic violence. The strength of emotions linked to social and family relationships, power relations and forms of control (emotional and psychological domination), are challenging to control during an interview, even when confidentiality and anonymity are guaranteed. Socio-cultural conceptions deeply rooted in social organization, and the regulation of social relations can act as significant barriers to the disclosure of violence. As a result, the statistical representation of violence in this survey likely reflects a conservative estimate, despite the efforts made during data collection to build trust and encourage open discussion. Fourth, recall bias may have influenced the findings, particularly among older participants who may have had difficulty accurately recalling distant experiences. Fifth, sampling limitations may have affected the completeness of the findings. The sample included individuals living in “*ordinary*” households but excluded those from “*collective*” households (e.g., medersas, *daaras*, educational institutions), as well as some highly vulnerable groups (such as individuals living on the street, minors deprived of guardianship, individuals with mentally illness, and beggars) who were excluded from the sample because of the violence perpetrated against them (stigmatization, discrimination, violence due to neglect) and may have contributed to the under-reporting of certain forms of violence. Sixth, there are conceptual challenges related to how certain forms of violence are understood, and therefore of the ability of actors to express them, particularly when they are in dissonance with experiences linked to the local context. It is worth questioning to what extent participants fully grasp the meaning of terms such as “*neglect-related violence*”, “*sexual violence*” or “*psychological violence*”, and what social representations they attached to these concepts, despite efforts by interviewers, who were trained to use sensitive follow-up questions to encourage disclosure and clarify responses. Neglect offers a paradigmatic example. Its unintentional and chronic nature contributes to its invisibility. In societies that have recently opened up to issues of mental health in their modern acceptation, neglect remains a social unthought, partly because it is not conceptualized and defined in the public and political space in charge of child rights and welfare, and partly because many acts of neglect are perceived as legitimate or acceptable from the point of view of the actors. The survey itself occupies a paradoxical position. On one hand it offered a cathartic and rare opportunity for participants to speak freely about sensitive and intimate subjects, which are often silenced or censored in social circles, within a structured and confidential setting. On the other hand, the standardized nature of survey instruments may inadvertently contribute to the invisibility of certain forms of violence by failing to capture culturally nuanced understandings or experiences that fall outside predefined categories. Given these limitations, future studies would benefit from incorporating qualitative or mixed methods approaches to fully capture the nuanced and culturally embedded dimensions of violence and its impact on mental health.

## Conclusion

This study highlighted the extent of violence in Senegal, its distribution according to region, gender and age, and its relationship with suicide risk. Given the level of prevalence observed, it is even more necessary/urgent to put in place a national plan to fight violence and a suicide prevention plan based on these initial epidemiological data, to take effective action against violence by identifying at-risk groups. The consequences of violence are still largely underestimated and understudied in Africa. If Senegal wishes to achieve target 5.2 of the SDGs, which focuses on eliminating violence against women and girls in order to expand globally [87], it will need to make additional efforts in various sectors (political, legal, health, economic). Similarly, reducing mortality from suicide has been prioritized by the WHO as a global goal and included as an indicator in the SDGs under target 3.4, as well as in the 13th WHO General Program of Work 2019–2023 and the WHO Mental Health Action Plan 2013–2020, which has been extended to 2030. Achieving this goal requires a comprehensive and coordinated suicide prevention response to ensure that suicide does not continue to cost lives and affect people’s well-being through the loss of loved ones or through suicide attempts.

With this in mind, awareness-raising and training are key challenges. In particular, we need to change the way we look at victims, by moving away from a culture of blame towards a culture of prevention and victim protection. Different categories of professionals in the health, social welfare, education, law enforcement and justice sectors need to acquire knowledge of the different types of violence and their social and psychological consequences, as well as skills in listening to and supporting victims, and in monitoring and evaluating the projects implemented. These action plans must be integrated into an appropriate legislative and legal framework, and the practical application of international conventions signed by Senegal to ensure respect for the rights of vulnerable groups. The prevention of violence and suicide cannot be achieved independently of investment in mental health, greater integration in primary health care, and greater decentralization in pursuit of the efforts undertaken in recent years.

A public health approach to violence prevention relies on the production of epidemiological data and data characterizing social determinants. This statistical approach would also benefit from being linked to research in anthropology or law to better understand the logics of actors at individual, community and institutional levels. The creation of a violence observatory linked to an anti-violence program would raise the profile and legitimacy of this issue within society and enable progress to be monitored for the benefit of all actors (victims, families, society).

## Supporting information

S1 Text Sample size calculation details. (DOCX)

S1 Checklist Inclusivity in global research. (DOCX)
