# Supplementary material for: Epidemiology of violence and suicide risk in Senegal: A nationwide survey in 2023
Source: PLOS Glob Public Health. 2026 Jan 2;6(1):e0005782. doi: 10.1371/journal.pgph.0005782 (PMC12758704; doi:10.1371/journal.pgph.0005782)
Supplement: S1 Text — (DOCX) [file pgph.0005782.s001.docx]

**S1 Text: Sample size calculation details**

| - Risk of alpha error : | 0,05 | $N=Z^{2}*\frac{p*\left( 1-p \right)}{e^{2}}*d*t*s*a$ |
| --- | --- | --- |
| - Standard error (Z) : | 1,96 |  |
| - Prevalence of violence in Senegal (p) (unknown) : | 0,5 |  |
| - Margin of error (e) : | 5% |  |
| - Cluster effect (d) : | 2 | $N={1,96}^{2}*\frac{0,5*\left( 1-0,5 \right)}{{0,05}^{2}}*2*0,2*2*6=1843$ individuals |
| - Non-response rate (t) : | 20% |  |
| - Age strata (a) : | 6 |  |
